# Integrating Precision Medicine and Digital Health in Personalized Weight Management: The Central Role of Nutrition

**DOI:** 10.3390/nu17162695

**Published:** 2025-08-20

**Authors:** Xiaoguang Liu, Miaomiao Xu, Huiguo Wang, Lin Zhu

**Affiliations:** 1 College of Sports and Health, Guangzhou Sport University, Guangzhou 510500, China; liuxg@gzsport.edu.cn (X.L.); 11450@gzsport.edu.cn (H.W.); 2 Research Center for Innovative Development of Sports and Healthcare Integration, Guangzhou Sport University, Guangzhou 510500, China; 3 College of Physical Education, Guangdong University of Education, Guangzhou 510800, China; miaomiaoxu@gzucm.edu.cn; 4 School of Physical Education and Health, Guangzhou University of Chinese Medicine, Guangzhou 510006, China; 5 Innovative Research Center for Sports Science in the Guangdong-Hong Kong-Macao Greater Bay Area, Guangzhou Sport University, Guangzhou 510500, China

**Keywords:** precision nutrition, personalized diet, nutrigenomics, metabolomics, microbiome, obesity, digital health, omics integration

## Abstract

Obesity is a global health challenge marked by substantial inter-individual differences in responses to dietary and lifestyle interventions. Traditional weight loss strategies often overlook critical biological variations in genetics, metabolic profiles, and gut microbiota composition, contributing to poor adherence and variable outcomes. Our primary aim is to identify key biological and behavioral effectors relevant to precision medicine for weight control, with a particular focus on nutrition, while also discussing their current and potential integration into digital health platforms. Thus, this review aligns more closely with the identification of influential factors within precision medicine (e.g., genetic, metabolic, and microbiome factors) but also explores how these factors are currently integrated into digital health tools. We synthesize recent advances in nutrigenomics, nutritional metabolomics, and microbiome-informed nutrition, highlighting how tailored dietary strategies—such as high-protein, low-glycemic, polyphenol-enriched, and fiber-based diets—can be aligned with specific genetic variants (e.g., FTO and MC4R), metabolic phenotypes (e.g., insulin resistance), and gut microbiota profiles (e.g., *Akkermansia muciniphila* abundance, SCFA production). In parallel, digital health tools—including mobile health applications, wearable devices, and AI-supported platforms—enhance self-monitoring, adherence, and dynamic feedback in real-world settings. Mechanistic pathways such as gut–brain axis regulation, microbial fermentation, gene–diet interactions, and anti-inflammatory responses are explored to explain inter-individual differences in dietary outcomes. However, challenges such as cost, accessibility, and patient motivation remain and should be addressed to ensure the effective implementation of these integrated strategies in real-world settings. Collectively, these insights underscore the pivotal role of precision nutrition as a cornerstone for personalized, scalable, and sustainable obesity interventions.

## 1. Introduction

Obesity constitutes one of the most urgent public health concerns worldwide, substantially elevating the risk of chronic conditions such as type 2 diabetes, cardiovascular disease, and non-alcoholic fatty liver disease [1]. Alarmingly, global obesity prevalence continues to rise across age groups, emphasizing the need for more effective and personalized intervention strategies [2]. Traditional weight management programs—typically based on standardized dietary and exercise prescriptions—often fail to accommodate the heterogeneity in individuals’ genetic profiles, metabolic pathways, microbiome composition, and dietary responses [3,4]. This “one-size-fits-all” approach leads to poor adherence, weight regain, and suboptimal long-term outcomes [5].

As a response, the integration of precision medicine and digital health tools is emerging as a transformative paradigm in obesity management [6]. Our primary goal is to identify and explore the most influential biological and behavioral factors relevant to precision medicine, particularly in relation to nutrition, while also discussing how these factors can be integrated into digital health platforms. Thus, we aim to focus on both the biological components and their integration into digital health tools. Precision medicine enables the personalization of interventions by leveraging multi-omics data (e.g., genomics, metabolomics, and microbiome profiling) [7], while digital health platforms allow for continuous monitoring and dynamic feedback [8].

A key pillar in this personalized framework is nutrition. Nutritional status and dietary intake are modifiable factors that interact with biological systems and significantly influence metabolic health [9]. Nutritional interventions remain a cornerstone of obesity treatment. Specific dietary components—such as dietary fiber, omega-3 fatty acids, polyphenols, and fermented foods—have been shown to modulate gut microbiota composition, reduce systemic inflammation, and improve metabolic flexibility [10,11]. For instance, dietary fibers promote the production of short-chain fatty acids (SCFAs), which improve insulin sensitivity and energy metabolism through gut–brain and gut–liver signaling pathways [12]. Likewise, omega-3 polyunsaturated fatty acids exert anti-inflammatory effects via nuclear factor kappa-B inhibition and lipid mediator pathways [13]. These mechanisms underpin the growing emphasis on precision nutrition, which—when informed by omics-based profiles and enhanced by real-time digital monitoring—offers an individualized dietary approach to optimize weight management outcomes [14].

Recent studies demonstrate that responses to specific diets (e.g., low-carbohydrate, high-fiber, or Mediterranean) can vary greatly between individuals based on genetic polymorphisms, insulin sensitivity, or gut microbial composition [15,16]. In parallel, mobile applications and wearable tools now allow users to log meals, monitor macronutrients, and receive personalized dietary guidance [17]. These digital tools are increasingly integrated with artificial intelligence and machine learning algorithms that adapt meal plans to user-specific needs and preferences [18,19].

However, many existing reviews examine either biological mechanisms or digital strategies in isolation and overlook the synergy between these two domains—particularly the role of precision nutrition as a converging link [20,21]. This review aims to bridge that gap by synthesizing evidence from the recent literature (2020–2025), proposing a unified framework, and identifying practical pathways for implementation. Specifically, this review aims to identify key biological and nutritional effectors influencing personalized weight control within the framework of precision medicine. To support practical translation, we also highlight their current or potential integration into digital health tools, including mobile applications, wearable devices, and AI-driven platforms for self-monitoring and feedback.

Although terms such as individualized, personalized, tailored, and precision nutrition (or weight management) are sometimes used interchangeably, they carry nuanced differences in the context of health interventions [4,7]. Individualized and tailored nutrition typically refer to interventions that are adapted based on a person’s stated preferences, lifestyle, or basic clinical parameters [22]. Personalized nutrition often incorporates broader personal data, including phenotype, behavior, and sometimes genetic information [14]. In contrast, precision nutrition represents the most comprehensive approach—it utilizes multi-omics data (genomics, metabolomics, and microbiome), digital biomarkers, and advanced analytics to inform and dynamically adjust interventions [6,7]. In this review, we adopt precision nutrition and precision weight management as core concepts, defined as the integration of omics-based profiling and digital feedback systems to deliver highly specific, dynamic, and evidence-driven strategies for optimizing weight outcomes [20].

## 2. Methods

This review provides a comprehensive synthesis of recent developments in the integration of precision medicine and digital health technologies for personalized weight management. A systematic literature search was conducted to identify relevant studies published from January 2020 to July 2025 using the following databases: PubMed, Web of Science, Scopus, and Google Scholar.

Search strategies combined controlled vocabulary (e.g., MeSH terms) and free-text keywords to identify relevant studies on precision medicine, digital health, and nutrition-related interventions in the context of obesity and weight management. The search was specifically aimed at identifying studies that explore the intersection of precision nutrition and digital health tools, with an emphasis on how omics-based profiles (genomics, metabolomics, and microbiome analysis) are integrated into digital platforms for personalized weight management. Key terms included “Precision Medicine”, “Personalized Nutrition”, “Personalized Medicine”, “Nutrigenomics”, “Nutritional Genomics”, “Dietary Patterns”, “Dietary Intervention”, “Genomics”, “Obesity”, “Weight Management”, “Metabolomics”, “Microbiome”, “Omics”, “Digital Health”, “Mobile Health”, “Wearable Technology”, “eHealth”, and “Artificial Intelligence” and their combinations with “Lifestyle Intervention”, “Digital Nutrition”, or “Nutritional Assessment”. Boolean operators (AND, OR) and truncation techniques were applied to optimize search sensitivity and specificity across databases such as PubMed, Web of Science, and Scopus. In addition, reference lists of included studies and recent systematic reviews were manually screened to identify further eligible publications related to precision nutrition, digital dietary monitoring, and personalized dietary strategies for obesity and metabolic health management.

Studies were included if they met the following criteria: (1) peer-reviewed publications in English; (2) studies conducted on human participants, including randomized controlled trials, observational studies, cohort studies, or systematic reviews; (3) focus on precision medicine, digital health, or their integration in the context of obesity or individualized weight management; and (4) relevance to genomics, metabolomics, microbiome modulation, digital intervention tools, AI-supported decision-making, or digital lifestyle monitoring. Exclusion criteria included non-English studies, preprints or grey literature, animal or in vitro studies, and those lacking relevance to the review objectives.

Although we limited our review to English-language publications, we acknowledge that relevant studies may also exist in other languages such as Chinese, Russian, French, or German. Since many non-English publications include English abstracts, this language restriction may have excluded some valuable evidence and should be considered a potential limitation of this study.

**Figure 1:**
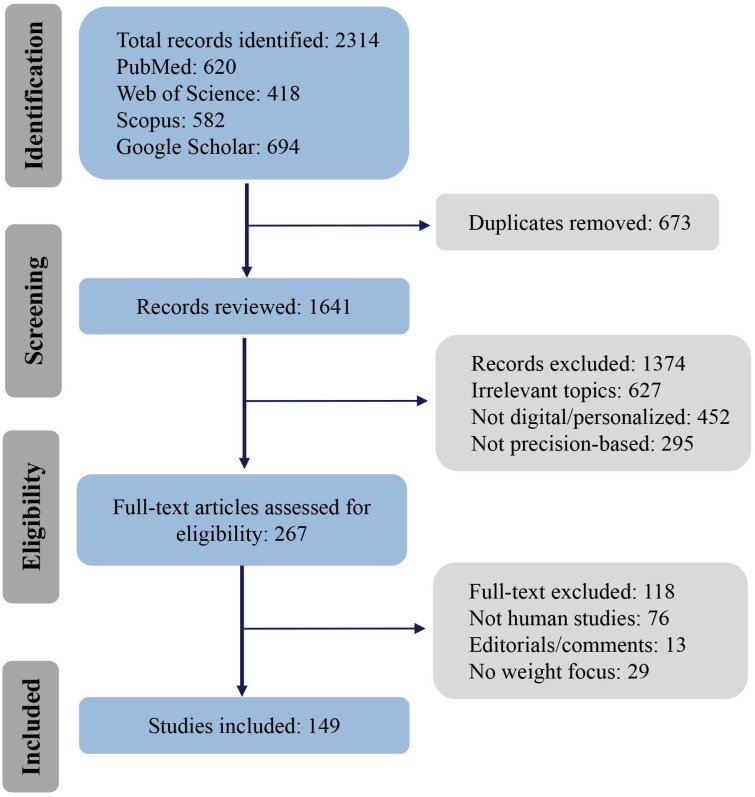
Literature Selection Flow Diagram.

A total of 149 articles were identified and screened based on relevance and inclusion criteria. The screening process involved an initial review of titles and abstracts, followed by full-text screening to confirm eligibility. A PRISMA-like flow diagram outlining the study selection process is provided below to increase transparency (Figure 1).

## 3. Biological Basis of Precision Medicine in Weight Management

### 3.1. Genetics and Obesity

The genetic susceptibility to obesity has long been confirmed by research [23,24]. Through genomic analysis, scientists have identified multiple genes associated with obesity, including the fat mass and obesity-associated gene (FTO), melanocortin 4 receptor gene (MC4R), and leptin gene (LEP) [25,26,27]. These genes directly affect an individual’s weight by regulating appetite, energy balance, fat accumulation, and metabolic rate [28,29].

Importantly, genetic profiles also modulate individuals’ dietary responses. For instance, individuals with FTO-risk alleles tend to have elevated appetite and lower satiety responses, indicating the need for personalized dietary interventions such as high-protein, low-glycemic index diets [30,31]. Several studies have shown that gene–diet interactions significantly influence weight loss outcomes, with certain genetic variants associated with differential responses to low-fat or Mediterranean diets [32,33,34].

**Figure 2:**
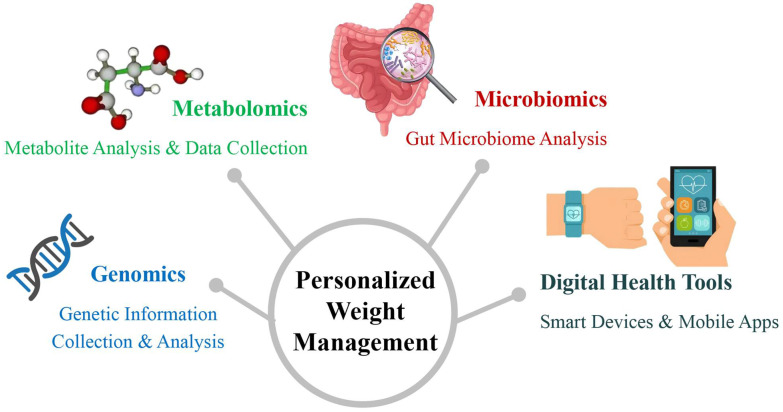
Precision Medicine Model for Personalized Weight Management. This figure illustrates how precision medicine integrates genomics, metabolomics, microbiomics, and digital health tools to achieve personalized weight management. By collecting individual genetic information, metabolic traits, and gut microbiome data and combining them with real-time monitoring and interventions through smart devices and mobile apps, personalized weight management plans can be provided for each person, leading to more precise and effective health management.

Therefore, precision medicine can use genomic testing to identify an individual’s susceptibility to obesity and provide tailored intervention strategies [35,36] (Figure 2). Personalized nutrition recommendations based on genotyping may include diet composition adjustments, caloric targets, and nutrient timing strategies to mitigate genetic predispositions [37,38].

To further understand how individual biological profiles influence weight regulation, it is essential to explore downstream metabolic processes, which are captured through metabolomics approaches.

### 3.2. Metabolomics: A New Perspective on Weight Management

Metabolomics offers a new perspective by helping us deeply understand the metabolic mechanisms of obesity [39]. Through comprehensive analysis of metabolic products in the body, metabolomics can reveal an individual’s metabolic characteristics and how different diets and exercises affect metabolic states [40,41].

Emerging nutritional metabolomics research demonstrates that metabolite signatures (e.g., branched-chain amino acids and lipid intermediates) can predict individual responses to dietary patterns [42,43]. For example, elevated baseline levels of branched-chain amino acids have been associated with insulin resistance and may predict poorer responses to high-carbohydrate diets but improved outcomes with higher-protein or lower-glycemic-load interventions [13,44].

These metabolic data enable the creation of personalized intervention strategies. For instance, individuals with impaired lipid oxidation have shown better weight outcomes following moderate-intensity aerobic programs [45], while those with better metabolism may focus more on strength training and moderate caloric intake [46].

Metabolomics-based personalized nutrition is increasingly supported by digital platforms that integrate metabolite profiling with dietary tracking and real-time feedback [4,47]. This allows for continuous optimization of nutritional interventions.

Beyond metabolic signatures, another emerging and critical factor in personalized weight management is the composition and function of the gut microbiome, which serves as the interface between host biology and diet.

### 3.3. The Role of the Microbiome in Obesity

**Figure 3:**
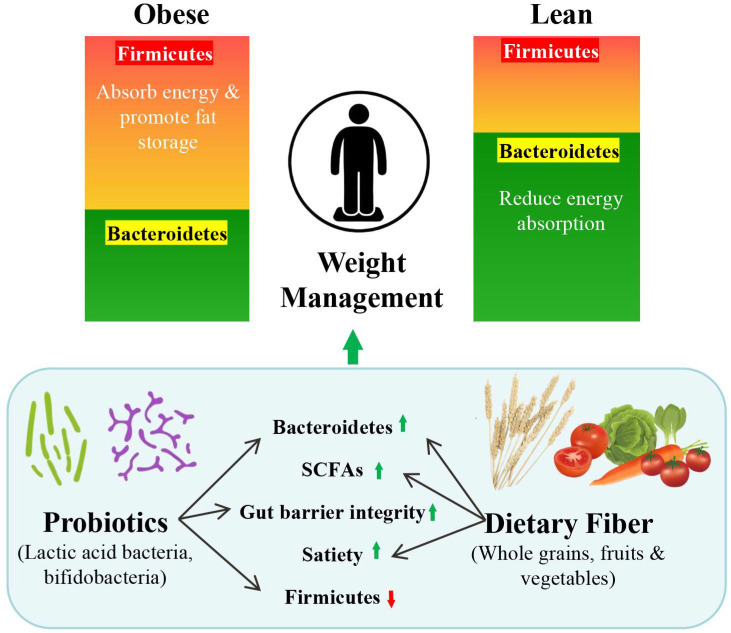
Role of Gut Microbiota Modulation in Weight Management. This diagram illustrates the relationship between gut microbiota composition and weight management. Obese individuals have a higher ratio of Firmicutes to Bacteroidetes, promoting energy absorption and fat storage. In contrast, lean individuals exhibit a lower Firmicutes/Bacteroidetes ratio, reducing energy absorption. Dietary interventions such as probiotics (e.g., lactic acid bacteria and bifidobacteria) and dietary fiber (from whole grains, fruits, and vegetables) can increase Bacteroidetes abundance, enhance SCFA production, improve gut barrier integrity, and promote satiety, ultimately contributing to weight management. SCFA, short-chain fatty acid.

The gut microbiome plays a crucial role in the occurrence and development of obesity [48,49,50]. Recent studies have found that the gut microbiota of obese individuals differs significantly from that of non-obese individuals [51,52,53] (Figure 3). In obese individuals, the gut microbiota often shows an elevated Firmicutes-to-Bacteroidetes ratio [54,55,56], which is associated with higher energy extraction and fat deposition [57,58].

Microbiome-informed nutrition is an emerging approach within precision nutrition [59]. For instance, individuals with low microbial diversity or low abundance of *Akkermansia muciniphila* may benefit from polyphenol-rich foods, prebiotics, or fermented foods [60,61].

Short-chain fatty acids (SCFAs) produced by microbial fermentation of dietary fibers also play a regulatory role by promoting the secretion of satiety hormones such as glucagon-like peptide-1 and peptide YY [62,63]. Therefore, dietary interventions including high-fiber intake, resistant starch, or probiotics can modify the gut microbiome to promote weight loss [64,65].

Digital health platforms are increasingly incorporating microbiome data to generate personalized dietary recommendations [66]. These tools use microbiota sequencing and food-response algorithms to suggest microbiome-supporting meal plans, forming a feedback loop between microbial state and dietary behavior [67].

### 3.4. Summary of Biological Foundations in Precision Weight Management

In summary, precision medicine in weight management integrates insights from multiple biological systems. Genetic profiling reveals innate predispositions and helps tailor dietary and behavioral interventions [24]. Metabolomics provides a dynamic readout of biochemical states, enabling the design of responsive nutritional plans based on real-time data [41]. The gut microbiome, as a modifiable interface between diet and host metabolism, offers another layer of personalization through dietary modulation and probiotic strategies [49,68]. These three biological domains—genomics, metabolomics, and microbiomics—are not isolated but interact dynamically to shape individual responses to weight management interventions [39]. Thus, a systems biology approach supported by digital technologies is essential for effective and personalized obesity care.

## 4. Digital Health Tools for Precision Weight Management

### 4.1. Smart Health Devices and Personalized Monitoring

With the rapid development of digital health tools, wearable devices (such as smartwatches, fitness trackers, etc.) have become indispensable in weight management [69,70] (Figure 2). These devices can monitor an individual’s activity level, heart rate, calorie expenditure, sleep quality, and other physiological data in real time and help individuals assess their health status through data analysis [71,72]. Unlike traditional weight management methods, smart health devices provide personalized data support, enabling real-time adjustments to exercise intensity, frequency, and dietary strategies to achieve the best weight loss results [73,74]. Smart devices can also improve adherence—a recent meta-analysis reported a 17% higher compliance rate among wearable users [75].

Research shows that high-intensity interval training can significantly increase energy expenditure and fat metabolism [76,77]. With monitoring by smart devices, individuals can adjust the intensity and duration of their training based on real-time data, thereby optimizing the weight management process [78].

To provide a structured overview of current technologies, Table 1 summarizes representative platforms and tools for precision weight management, detailing their intervention forms, advantages, and key references. This integration of biological and digital tools provides a robust foundation for personalized intervention strategies [79].

However, it is important to acknowledge that individuals with severe obesity or related comorbidities may face substantial physical limitations, making high-intensity training impractical or unsafe [46]. In these cases, digital health tools should support low-impact, individualized activities—such as walking, aquatic exercise, or chair-based workouts—based on functional capacity assessments [69,80]. Adaptive exercise planning enabled by wearable devices can help users progress gradually, maintain motivation, and reduce injury risk [81,82]. Addressing these real-world constraints is crucial to ensure that precision exercise strategies are inclusive and equitable for those most in need of support.

**Table 1:** Representative Technologies in Precision Weight Management.

Technology Type	Representative Tools	Intervention Form	Advantages	Limitations	References
Genomics	23andMe, DNAfit (√)	Personalized diet and exercise plans	Individualized guidance	High cost; limited generalizability across populations	[83]
Metabolomics	Serum profiling (△)	Nutrition adjustment	Real-time feedback	Laboratory-dependent; low accessibility in remote areas	[84]
Microbiomics	Viome, 16S rRNA (△)	Probiotic/dietary adjustment	Improves gut health	Expensive; complex interpretation; limited standardization	[85]
Digital Tools	MyFitnessPal, wearables (√)	Self-tracking, coaching	Boosts adherence	Digital divide; limited access in low-income populations	[79]

Note: Symbols indicate the level of integration into digital health tools: √ = already integrated; △ = partially integrated or under development.

### 4.2. BCAAs and Muscle Damage

Mobile health applications—such as MyFitnessPal (featuring barcode scanning and automatic macronutrient analysis) and Lose It! (offering AI-powered meal logging and calorie prediction)—have become essential tools in daily weight management [86,87]. A 2022 systematic review and meta-analysis of 34 randomized controlled trials evaluated smartphone-based diet and lifestyle interventions. These included commonly used apps such as MyFitnessPal and were compared to usual care or active controls. Participants achieved an average weight loss of –1.99 kg at 3 months (95% CI: –2.19 to –1.79 kg) and –2.80 kg at 6 months (95% CI: –3.03 to –2.56 kg), highlighting the short-term efficacy of app-based dietary self-monitoring [88]. These apps allow users to log their intake, monitor nutrients and calories, and receive real-time nutritional feedback [89,90].

Advances in big data analytics and AI have enabled mobile apps to not only assess dietary patterns but also generate personalized meal plans based on individual metabolic profiles [91,92]. For individuals with lower metabolic efficiency, such plans may emphasize low-carbohydrate, high-protein diets, while those with higher efficiency may be guided to increase healthy fat intake [93,94]. These personalized dietary strategies enhance alignment with individual needs and have been shown to improve weight management outcomes, as supported by recent clinical evidence [80,82,95,96,97]. Table 2 summarizes key trials and reviews demonstrating improvements in weight loss, metabolic markers, and behavioral adherence through the use of smartphone apps and wearable devices.

While the average short-term weight loss (~2 kg in 3 months) reported in some app-based interventions may appear modest, especially for individuals with severe obesity (e.g., BMI ≥ 40), it should not be dismissed [93,94]. Clinical evidence suggests that even a 3–5% reduction in body weight—equivalent to 4–7 kg in a 140 kg person—can yield significant metabolic benefits, including improved glycemic control, blood pressure, and lipid profiles [35]. Moreover, digital tools may serve as low-barrier, scalable entry points to initiate behavioral change and promote adherence, particularly among individuals who may face physical, psychological, or financial barriers to intensive clinical programs [80,82]. As such, app-based interventions should be viewed as complementary tools that help bridge gaps in care and lay the foundation for more sustained and comprehensive weight loss efforts over time.

**Table 2:** Summary of Recent Clinical Studies Applying Digital Tools in Obesity Management.

Design & Sample	Intervention	Duration	Main Findings	References
RCT; N = 150 adults with obesity (BMI ≥ 30)	Multimodal app (“zanadio”) (√)	12 months	Mean weight loss 7.75% (95% CI –9.66 to –5.84); improvements in waist-to-height ratio and QoL.	[95]
RCT; N = 168 adults with BMI 30–40	Multimodal app (ADHOC) vs. delayed access (√)	12 weeks + 12-week follow-up	Greater short-term weight reduction and improved dietary intake and QoL.	[96]
Systematic review and component network meta-analysis; includes 68 RCTs	Digital support features across weight-loss apps (√)	Up to 12 months	Identified key components (education; specialist contact) associated with weight loss (–2.52 kg at 6 months; –2.11 kg at 12 months).	[80]
Umbrella review; 507 RCTs (N ≈ 206,873)	Digital health (apps, wearables, and SMS) (√)	Mostly 3–6 months	Modest but significant improvements: weight (–1.89 kg), steps/day (+1329), sedentary behavior, and energy intake (–103 kcal/day).	[97]
Cohort study; N = 46,579 adults	Wearables vs. pedometer apps (√)	12–24 weeks	Wearable users showed higher physical activity, improved diet, and reduced metabolic syndrome risk.	[81]
Observational real-world study; N = 2217 CGM users	CGM + wearables + app-based coaching (√)	28 days	Reduced caloric intake, increased activity, and improved glycemic and metabolic outcomes.	[82]

BMI: body mass index; RCT: randomized controlled trial; CI: confidence interval; QoL: quality of life; SMS: short message service; CGM: continuous glucose monitoring. Note: Symbols indicate the level of integration into digital health tools: √ = already integrated.

### 4.3. Data Sharing and Cross-Platform Collaboration

With the accumulation of health data and the widespread use of digital health tools, data sharing and cross-platform collaboration have become possible [98,99]. Individuals can share their exercise, diet, and sleep data with doctors, nutritionists, and health management experts through health applications, enabling cross-disciplinary collaboration to create more comprehensive weight management plans [100,101].

Data sharing not only promotes the implementation of personalized interventions but also provides valuable sample data for scientific research, helping to further validate the effectiveness of precision weight management methods [102]. However, future implementation requires alignment with relevant data privacy and protection frameworks, which may impose limitations on real-time sharing and secondary use of personal health information [103,104]. Ensuring compliance with such regulations is essential for secure and ethical cross-platform data utilization [105,106].

**Figure 4:**
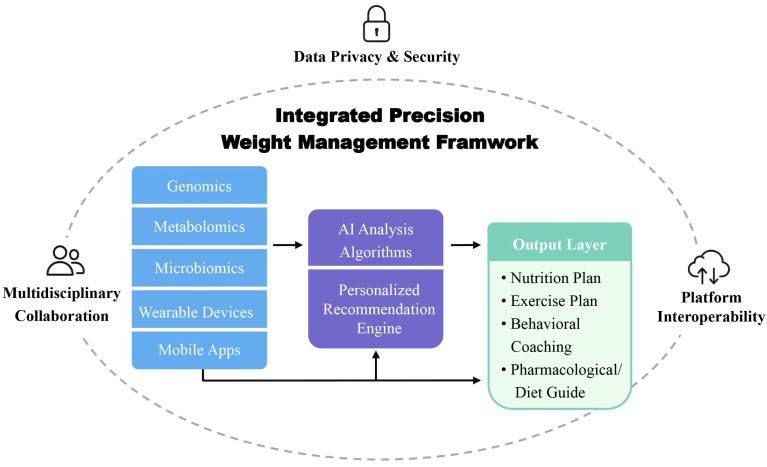
Integrated Precision Weight Management Framework. This diagram presents a multi-layered framework for precision weight management that integrates biological data and digital technologies. The input layer includes individual-level genomics, metabolomics, and microbiomics, alongside real-time lifestyle data collected via wearable devices and mobile health applications. These data are processed through artificial intelligence algorithms and a personalized recommendation engine in the intermediate layer. The output layer provides individualized intervention strategies, including nutrition and exercise plans, behavioral coaching, and pharmacological or nutritional guidance. The entire system is supported by a surrounding layer emphasizing data privacy and security, platform interoperability, and multidisciplinary collaboration, forming a closed-loop structure for continuous optimization of weight management outcomes.

A comprehensive integration of these technologies—ranging from omics-based personalization to real-time monitoring and data-driven recommendations—is illustrated in the precision weight management framework [107,108] (as shown in Figure 4). This model highlights how multi-source data can be translated into individualized interventions through AI-powered platforms, supported by data security and interdisciplinary collaboration [109].

### 4.4. Personalized Dietary Interventions in Precision Weight Management

#### 4.4.1. Dietary Strategies Based on Individual Phenotypes

Metabolic and hormonal heterogeneity influences individual responses to dietary interventions [4,110]. For example, individuals with insulin resistance may respond better to low-carbohydrate or ketogenic diets, while those with hyperinsulinemia or visceral obesity show greater benefit from Mediterranean or high-fiber diets [15,16]. A randomized trial in 2023 demonstrated that stratifying participants by insulin sensitivity led to significantly improved weight loss outcomes (−5.4 kg vs. −2.8 kg; *p* < 0.01) [111]. These findings support the use of phenotype-based dietary strategies. Mechanistic explanations are further discussed in Section 4.5.

#### 4.4.2. Macronutrient-Specific Precision Interventions

Macronutrient composition affects satiety, energy balance, and body composition [112]. High-protein diets help preserve lean mass and promote satiety, particularly in individuals with FTO-risk alleles or reduced ghrelin suppression [28]. Dietary fibers, such as resistant starch and β-glucans, improve glycemic control and support gut health. Polyphenol-rich foods may enhance metabolic flexibility by modulating microbiota composition [10,11,60,65]. The biological mechanisms behind these effects are detailed in Section 4.5.

#### 4.4.3. Gene–Diet Interactions and Nutrigenomics

Numerous gene–diet interactions have been identified in relation to weight regulation [113]. FTO A allele carriers tend to respond better to high-protein or Mediterranean diets than to low-fat diets [114,115]. Polymorphisms in genes such as MC4R and LEPR may influence appetite control and dietary response [116,117]. Commercial nutrigenetic tools like DNAfit and 23andMe offer gene-based recommendations, though further validation is needed [118,119]. The summary in Table 3 highlights emerging diet-based strategies tailored to individual biological profiles, supported by recent studies.

**Table 3:** Personalized Nutrition Interventions by Population, Mechanism, and Tools.

Intervention Type	Target Population	Mechanistic Rationale	Digital/Omics Tools	References
Low-Carbohydrate/Ketogenic Diet	Insulin-resistant individuals, prediabetes, and type 2 diabetes	Reduces insulin secretion and enhances fat oxidation	Continuous glucose monitor; activity tracker (√)	[120]
Green-Mediterranean Diet	Individuals with visceral obesity and chronic inflammation	Activates AMPK and short-chain fatty acid production; improves body composition	16S rRNA sequencing; MRI (△)	[121]
Resistant Starch Supplementation	Individuals with low gut microbiota diversity	Increases SCFA production; supports satiety hormone signaling	Metagenomics; targeted metabolomics (△)	[65]
Digital Nutrition Feedback System	Individuals with poor dietary adherence or metabolic risk	Improves self-regulation via real-time glucose and activity feedback	App-based monitoring system; wearable sensors (√)	[82]
Genotype-Based Dietary Advice	Carriers of FTO- or polygenic-risk alleles	Aligns macronutrient ratios with genetic response patterns	Genetic risk profiling; nutrigenomics (△)	[102]

Note: Symbols indicate the level of integration into digital health tools: √ = already integrated; △ = partially integrated or under development.

### 4.5. Mechanistic Foundations of Nutritional Interventions in Personalized Weight Managementt

#### 4.5.1. Gut–Brain Axis and Hormonal Satiety Signaling

Dietary components such as fiber, medium-chain triglycerides, and amino acids can stimulate anorexigenic gut hormones including GLP-1, peptide YY (PYY), and cholecystokinin (CCK), which regulate appetite via hypothalamic pathways [122,123]. These hormonal responses are often enhanced in individuals consuming high-protein or low-glycemic-index diets [124].

#### 4.5.2. Microbiota-Driven Fermentation and Energy Balance

Fermentable fibers are converted by gut microbes into SCFAs such as acetate, propionate, and butyrate [125]. SCFAs help maintain gut barrier function, modulate inflammation, and promote satiety by activating G-protein-coupled receptors [126]. Diets that increase *Akkermansia muciniphila* or *Faecalibacterium prausnitzii* abundance have been associated with improved metabolic flexibility and reduced visceral fat [127,128].

#### 4.5.3. Molecular and Genomic Modulation of Metabolism

Precision diets can modulate pathways related to lipid oxidation, insulin sensitivity, and adipogenesis [129]. Polyphenol intake may activate AMP-activated protein kinase (AMPK) and downregulate sterol regulatory element-binding protein 1c (SREBP-1c) and peroxisome proliferator-activated receptor gamma (PPAR-γ), supporting lipid mobilization [130]. These mechanisms may explain improved outcomes in FTO- and MC4R-risk allele carriers on protein-rich or Mediterranean diets [115]. Dietary patterns can also influence DNA methylation of obesity-related genes, providing a potential epigenetic basis for long-term weight regulation [131].

#### 4.5.4. Anti-Inflammatory Pathways and Adipokine Regulation

Obesity is often accompanied by chronic low-grade inflammation [132]. Anti-inflammatory nutrients, including omega-3 fatty acids, curcumin, and polyphenols, may reduce cytokine expression and improve adipokine profiles, thereby supporting metabolic homeostasis [133]. These interventions may be particularly beneficial when combined with digital tools for inflammation monitoring [134].

#### 4.5.5. System-Level Integration with Digital Platforms

**Figure 5:**
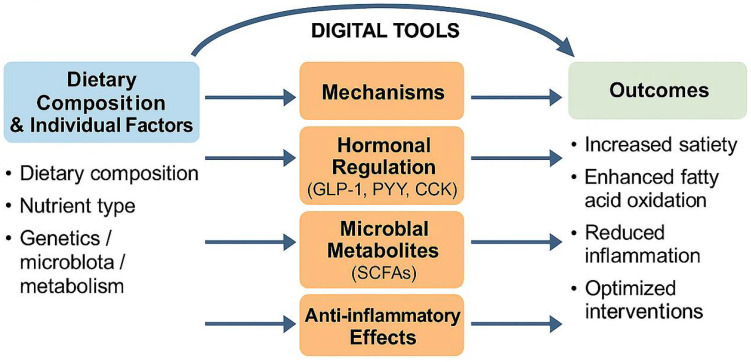
Mechanistic Pathways of Personalized Nutrition Interventions in Weight Management. This conceptual diagram illustrates five interconnected biological pathways through which personalized nutrition contributes to weight control and metabolic health. These include (1) hormonal regulation of appetite via the gut–brain axis, where nutrients modulate GLP-1, PYY, and CCK secretion; (2) fermentation of dietary fibers into SCFAs that promote energy balance through GPR signaling; (3) gene–diet and epigenetic interactions that influence metabolic flexibility (e.g., via AMPK and PPAR-γ); (4) suppression of chronic low-grade inflammation through anti-inflammatory nutrients that reduce CRP, IL-6, and TNF-α levels while enhancing adiponectin secretion; and (5) real-time digital feedback loops integrating physiological biomarkers (e.g., CGM, microbiome, and wearables) to support individualized and adaptive dietary strategies. These mechanisms are synergistic and modulated by individual omics profiles and behavior, enabling precision-guided intervention in weight control programs.

Digital tools such as continuous glucose monitors (CGM), wearable devices, and microbiome sequencing enable real-time tracking of physiological responses [82]. AI-based feedback systems can use these data to tailor dietary guidance and improve adherence [109]. Such integration bridges biological mechanisms with personalized behavior change (Table 4) (Figure 5).

Despite the growing potential of AI-driven personalization, developing a valid and generalizable model requires access to large, diverse datasets with well-characterized variables [21,109]. These include sex, ethnicity, age, metabolic markers, genetic profiles, lifestyle data, and longitudinal outcome measures. For example, real-world training datasets may require thousands of participants from different demographic backgrounds to avoid algorithmic bias [106,135]. Furthermore, the cost of developing and deploying such models—including data acquisition, processing, and model validation—can be substantial. While early implementations may rely on academic or governmental funding, future scalability may depend on public–private partnerships or integration into national health platforms to ensure affordability and accessibility [105].

**Table 4:** Mechanistic Summary of Personalized Nutrition Interventions.

Pathway	Key Mechanisms	Target Biomarkers	Representative Interventions	References
Gut–Brain Axis	Secretion of satiety hormones (GLP-1, PYY, and CCK) that modulate hypothalamic appetite control	Post-prandial GLP-1/PYY, fasting insulin, and ghrelin	High-protein/low-GI snacks (e.g., tree-nut inclusion)	[136]
Microbiota–SCFA	Fermentation of prebiotic fibers into SCFAs which activate GPR43/41, enhance gut barrier, and raise energy expenditure	Fecal/plasma SCFAs, α-diversity, and *A. muciniphila* abundance	Inulin or resistant starch supplementation	[12]
Nutrigenomics	Diet-driven modulation of gene expression (AMPK; PPAR-γ) and epigenetic marks according to omic phenotype	FTO, MC4R variants; DNA-methylation profiles	Polyphenol-rich or genotype-matched meal plans	[4]
Inflammatory Modulation	Anti-inflammatory nutrients rebalance adipokines and lower CRP/IL-6/TNF-α	CRP, IL-6, TNF-α, and adiponectin	Curcumin (with piperine) 1 g·d^−1^	[137]
Digital Feedback Loop	Real-time data (CGM; wearables) drive AI-guided adjustment of diet and activity prescriptions	CGM metrics, step count, and adaptive macronutrient targets	mHealth app + self-experimentation protocol	[138]

GLP-1: glucagon-like peptide-1; PYY: peptide YY; CCK: cholecystokinin; GI: glycemic index; SCFAs: short-chain fatty acids; GPR: G-protein-coupled receptor; AMPK: AMP-activated protein kinase; PPAR-γ: peroxisome proliferator-activated receptor gamma; CRP: C-reactive protein; IL-6: interleukin-6; TNF-α: tumor necrosis factor alpha; CGM: continuous glucose monitoring.

## 5. Challenges and Future Prospects

Despite the promising potential of precision medicine and digital health tools in weight management, several challenges must be addressed [139]. These challenges can be categorized into technical, data-related, and ethical–policy dimensions.

### 5.1. Technical Challenges

The widespread adoption of genomics, metabolomics, and other precision technologies continues to face high costs and technical barriers, limiting their use in routine clinical or public health settings [140]. Advances in biotechnology and engineering are needed to reduce costs, improve scalability, and make these tools more accessible to the general population [141].

Moreover, several promising biological mechanisms—such as epigenetic regulation, microbiota-derived SCFA signaling, and nutrient–gene interaction pathways—have not yet been widely implemented in digital health platforms [60,123,131]. Barriers to their integration include the need for real-time biomarker detection, high cost of omics-based assays, lack of standardized measurement tools, and insufficient translation from laboratory research to user-friendly technologies [7,142]. Addressing these gaps will be essential to fully realize the potential of precision weight management.

### 5.2. Data Challenges

Privacy and security concerns regarding health data pose a major obstacle to the effective use of digital health tools [143]. Beyond safeguarding individual privacy and data security, challenges also include data interoperability across platforms and systems, which limits seamless integration and coordinated care [142].

### 5.3. Ethical and Policy Challenges

Issues such as informed consent, user authorization, and equitable access must be addressed. Without clear regulatory frameworks and ethical guidelines, the risk of misuse or unequal benefit distribution of precision tools may increase [144]. It is essential to establish transparent policies and legal safeguards to guide the responsible development and deployment of precision weight management technologies [135].

### 5.4. Future Prospects

Looking ahead, with continued advances in artificial intelligence, big data analytics, and digital infrastructure, precision weight management is expected to undergo major breakthroughs [145]. Integrated health platforms that combine wearable sensor data, gut microbiome profiling, and AI-powered recommendation engines may become the core of intelligent health coaching systems [146]. These platforms can incorporate biomarker monitoring, personalized interventions, and intelligent remote management to deliver real-time, tailored feedback on nutrition, exercise, and lifestyle [147]. Such systems will enable sustainable weight management in a personalized and automated manner [148]. This transformation not only enhances the effectiveness and scalability of weight control strategies but also offers new solutions to the global obesity crisis, paving the way for precision weight management to become a cornerstone of public health worldwide [149].

An integrated approach combining medical treatment and the proposed personalized strategies may offer additional benefits for individuals with obesity, particularly those with severe obesity or obesity-related complications [5,35]. For example, initiating treatment with approved anti-obesity medications [35] or metabolic surgery [5] could induce a more rapid and substantial initial weight loss, potentially enhancing mobility, physical function, and psychological readiness for sustained lifestyle changes [5,45]. Following this initial phase, the application of precision nutrition [4,102], digital health tools [8,22], and behavioral interventions [138,148] could help maintain weight loss and prevent relapse. This sequential strategy may maximize both short-term and long-term outcomes; however, it requires careful consideration of patient eligibility, cost, accessibility [106,149], potential side effects [5,35], and long-term adherence [5,124].

In this context, several biological effectors identified in this review—such as gut microbial diversity, insulin sensitivity, and specific genetic variants (e.g., FTO and MC4R)—have been frequently examined across multiple studies and showed consistent associations with weight outcomes [25,31,127]. Although a formal meta-analysis was not performed due to scope limitations, these high-frequency effectors represent promising candidates for future quantitative synthesis. Meta-analyses focused on these effectors could help validate their predictive utility, quantify effect sizes, and support the development of evidence-based digital health applications [37,38].

### 5.5. Equity and Accessibility Considerations

While longitudinal updates of omics data such as metabolomic or microbiome profiles would theoretically enhance the precision of weight management interventions, their routine use remains constrained by high cost, technical complexity, and limited accessibility [39,107]. To improve feasibility in real-world settings, future approaches may consider periodic rather than continuous biomarker monitoring (e.g., every 3–6 months), integration of surrogate clinical markers (e.g., waist-to-height ratio, fasting insulin, or C-reactive protein), and use of scalable digital tools for behavioral tracking [82,111]. In the early stages, such high-tech interventions may be more feasible in high-risk populations or institutional pilot programs, with potential for broader adoption as technologies mature and become more affordable [143].

## 6. Conclusions

This review systematically synthesized current advances in precision medicine and digital health technologies for individualized weight management, with a central emphasis on the role of nutrition and dietary interventions. By integrating multi-omics data—including genomics, metabolomics, and gut microbiome profiles—with digital tools such as mobile apps and wearable devices, precision nutrition enables personalized dietary strategies while supporting sustainable behavior change through real-time monitoring and adaptive feedback.

Looking ahead, the development of precision nutrition should prioritize the construction of interoperable digital nutrition platforms, the validation of individualized dietary interventions across diverse populations, and the transition from generalized dietary guidelines to personalized nutritional prescriptions. Additionally, ethical frameworks and data privacy regulations specific to nutrition-related information must be strengthened to ensure safe and equitable implementation. As technology and practice continue to advance, personalized nutrition is poised to become a key pillar in combating obesity and metabolic diseases, both in clinical settings and at the population level.

## Data Availability

All relevant materials are presented in this manuscript.
